# Sedentary Behavior Patterns of the Hungarian Adult Population

**DOI:** 10.3390/ijerph20032702

**Published:** 2023-02-02

**Authors:** Éva Bácsné Bába, Anetta Müller, Christa Pfau, Renátó Balogh, Éva Bartha, György Szabados, Zoltán Bács, Kinga Ráthonyi-Ódor, Gergely Ráthonyi

**Affiliations:** 1 Institute of Sports Economics and Management, University of Debrecen, 4032 Debrecen, Hungary; 2 Institute of Accounting and Finance, University of Debrecen, 4032 Debrecen, Hungary

**Keywords:** IPAQ, NCDs, physical inactivity, SBQ, sedentary lifestyle, sitting time

## Abstract

Background and aim: Nowadays, a high level of sedentary behavior (SB) is an important health issue. Many studies have focused on evaluating the physical activity (PA) level, while evaluation of SB has received less attention. The main goal of the present study is to describe the sedentary lifestyle of the Hungarian adult population and identify the vulnerable groups with high amount of sitting time and the patterns of SB. Another aim of this study is to compare the two types of questionnaires (International Physical Activity Questionnaire—IPAQ and Sedentary Behavior Questionnaire—SBQ) related to sitting time. Methods: This study analyzed cross-sectional primary data using self-reported questionnaires collected by a Hungarian research market company among the adult population in Hungary. The final sample of this study consisted of 1295 participants with a mean age of 45.9 years (SD = 15.2). Analysis of variance (ANOVA) test with post-hoc (Tukey) analysis were used to analyze the link between sitting time and socio-demographic variables (sex, age, BMI, settlement type, education level, marital status, work category, working hours, employment status, sport activity) and body mass index (BMI). Results and conclusions: According to the SBQ, on average, Hungarians sit for 469.53 min per day (7.81 h) on weekdays and 421.25 min per day (7.01 h) on weekends, which suggested a significant difference compared to IPAQ data: 287.82 min per day (4.79 h) on weekdays and 224.30 min per day (3.73 h) on weekends. Young people (aged between 18 and 29) were reported to have the highest average sitting time, i.e., 545 min per day (more than 9 h), and are showing the highest prevalence (53%) of sitting at least 480 min (8 h) per day. Sitting workers also had a high average sitting time, i.e., 514.82 min per day, and a high prevalence (49.3%) of sitting at least 480 min (8 h) per day. People who live in the capital city had higher sitting time, especially on working days. Men sat longer than woman, i.e., 19 min on working days and 45 min on weekends. The most frequent sedentary activities are: working on computer (126 min) on working days and watching TV (130 min) on weekends. Our results clearly show that the self-report single-item measure (IPAQ) significantly underestimates sedentary time compared to the multi-item questionnaire (SBQ). We identified vulnerable groups with high sitting times: men, young adults, inhabitants of the capital city and sitting workers. Consequently, these groups should be continuously surveyed, and requires specific interventions and strategies that particularly counteract the increased sitting time.

## 1. Introduction

Nowadays, sedentary behavior (SB) is a significant issue for health, with more and more research studying sedentary time and SB [1,2,3,4,5]. High levels of SB are strongly correlated to the risk of premature death, cancer and chronic diseases such as cardiovascular disease [6,7,8], metabolic syndrome and type 2 diabetes [6,9,10,11,12,13], osteoporosis [14] and mental diseases [15]. Lack of physical activity (PA) increases the risk of obesity [16], which affects almost every system in the body [17]. Physical inactivity is a leading contributor to global mortality [18,19]. The World Health Organization (WHO) estimates that non-communicable diseases (NCDs) account for 74% of deaths globally and Europe is one of the most affected regions [20]. Independent of PA, high levels of SB are linked to negative health outcomes [2,21]. According to Poses-Ferrer et al. [5], daily sitting time may be an independent risk factor for various NCDs and physical activity has no effect on the relationships between sitting time and any of the outcomes. It is important to clarify that SB is not equivalent to physical inactivity (PI). PI is characterized as not reaching the levels of moderate to vigorous PA, while SB-related activities (in a sitting, reclining or lying posture) have lower metabolic expenditure than 1.5 Metabolic Equivalent Task (MET) [22,23]. Despite the clear evidence that PA lowers the risk of all-cause mortality as well as NCDs, only 1 in 10 Hungarian adults (18–64 years) met the World Health Organization (WHO) guidelines in 2018. A person is considered physically active if they achieve 150 min of moderate-intensity or 75 min of vigorous-intensity physical activity per week (or a combination of the two). In addition, muscle-strengthening exercises must be included at least two days per week [24]. In addition, there are adults from this small group who meet the guidelines but are otherwise sedentary [12,25]. Due to the technology revolution, more people are employed in low-activity occupations. A major factor in workers’ daily sedentary time is the significant amount of sedentary time spent at work [26]. People can spend three-quarters of the working day in SB [27], and Kazi et al. [28] suggested that if people spend more time sitting during the working day, they will spend more time sitting during their leisure time [29].

The first major wave of research on the effects of SB came in the early 2000s. At that time, the main focus was on the association between time spent watching television and various diseases [30,31,32,33,34,35]. These studies often used TV viewing time as a proxy marker for SB. Later, sitting time was linked to nine activities—watching television, playing computer/video games, sitting while listening to music, sitting and talking on the phone, doing paperwork or office work, sitting and reading, playing a musical instrument, doing arts and crafts and sitting and driving/riding in a car, bus or train. In conjunction with this, the Sedentary Behavior Questionnaire (SBQ) was developed, which measures the nine activities separately on weekdays and weekends. The strength of the SBQ compared to other sedentary time questionnaires (e.g., the IPAQ, which also asks about sedentary time) is that it is more detailed, and therefore, provides more accurate results, despite being based on self-report [36,37]. Several studies have used an accelerometer or another device to measure SB objectively [11,35,38,39]. Although objective measures have higher validity to asses SB, they cannot provide insight into the specific behaviors or contexts that contribute to total sedentary time [35,38]. In contrast, subjective measurements with lower validity for measuring SB can offer rich contextual information about when and where sedentary behavior is occurring [38]. In the early 2000s, a number of studies investigated the SB of the European [30,40,41,42], Australian [31,43,44] and American [45,46,47,48] populations. In the last 3–4 years, there has been a renewed interest in this subject, as evidenced by the publication of studies on the Turkish [49], Portuguese [50], Dutch [51], Catalan [5] and Israeli [52] populations. These studies focused on the analysis of adult populations. In contrast in Hungary, the “Health Behavior in School-Aged Children” (2002–2010) questionnaire survey was administered, so it was used only among the child population [53]. However, no study, to the best of our knowledge, has yet described the SB especially the sitting time measured by SBQ among the adult Hungarian population. Therefore, the main goal of the present study is to describe the sedentary lifestyle of the Hungarian adult population, in order to confront the population with the fact that it is not enough to lead an active lifestyle, but also to reduce sedentary time is important for maintaining health. A specific aim of this study is to identify vulnerable groups with high sitting time and to describe which patterns of the SB contribute to the greatest extent to the high sitting time in order to contribute the development of specific interventions and strategies that particularly counteract the increased sitting time.

## 2. Materials and Methods

### 2.1. Study Subject

This was a cross-sectional study based on anonymous self-reported questionnaires, which was distributed by a Hungarian research market company among the adult population in Hungary from 1st November 2017 to 31st January 2018. The study collected information about the physical activity, sedentary behavior, mental health and the sports habits of the Hungarian adult population applying widely used validated questionnaires. In our previous paper, we presented the results related to the physical activity of the Hungarian adult population [54]; however, the present manuscript focuses on the sedentary behavior of the Hungarians. 

### 2.2. Instrument

The online questionnaire asked participants about their general characteristics, PA and SB. General characteristics contained sex, age, settlement type, region, marital status, education and occupation, working hours, household income, sports activity, height and body weight. Body Mass Index (BMI) was calculated from the self-reported weight (kg) and height (cm) values and the widely used BMI classification for adults was applied: Underweight < 18.50; Normal range 18.50–24.99; Overweight: 25.00–29.99; Obese > 30 kg/m^2^ (WHO) [54]. Sedentary time were measured with the long-form International Physical Activity Questionnaire (IPAQ) and the Sedentary Behavior Questionnaire (SBQ) [36,52]. The IPAQ is designed to assess the physical activity undertaken across different domains and time spent sitting in the last 7 days [55]. The IPAQ scoring protocol was used to process IPAQ data. Physical activity responses were converted to metabolic equivalent task (MET) [55]. According to MET rates, three physical activity categories (low, moderate, high) were defined. In the present study, these categories were used to compare the sitting time in each group. The SBQ was adapted from Rosenberg et al. [37]. The main purpose of the questionnaire is to estimate sedentary time for nine different activities (watching television, playing computer/video games, sitting while listening to music, sitting and talking on the phone, doing paperwork or office work, sitting and reading, playing a musical instrument, doing arts and crafts and sitting and driving/riding in a car, bus or train) for weekdays and weekend days [37]. For the summary variables of total hours/day spent in SB (weekday and weekend) responses higher than 24 h/day were truncated to 24 h/day [37]. Based on the findings of the current scientific literature, sitting more than 480 min (8 h) a day is associated with increased risk of mortality [11,51,56]. Time spent sitting was dichotomized into sitting less than 480 min and sitting for at least 480 min. According to several studies [37,49,57], the overall reliability of the SBQ items and total scores was acceptable. The main advantage of the SBQ is that using questions about specific SB helps people to recall relevant information more precisely than asking about all SB [37]. In designing our questionnaire, we added some extra information to the items, indicating the info-communication devices in use today. For example, the television item has been supplemented with the following information: includes videos from any source, e.g., DVD, blu-ray, movie channel, video library, etc., while the computer games item has been supplemented with the following examples: video games, online games.

### 2.3. Data Collection

Data collection and data cleaning were carried out by a market research company (Szinapszis Market Research & Consulting Ltd., Debrecen, Hungary) via self-reported questionnaires with a mixed data collection methodology (computer-assisted web interviewing and computer-assisted telephone interviewing) in 2018. A total number of 1343 participants completed the questionnaire. With regard to age, participants had to be over the age of 18. The sample was representative in four characteristics: sex, age, type of settlement and region [54]. We considered it important to break down the data and present and focus the results in this way, because the lifestyle elements, including the SB, are determined and influenced by these variables. After data cleaning process included the guidelines of scoring protocols 1295 participants remained in the final database. 

### 2.4. Ethical Approval

The survey was designed to protect respondent anonymity. The study protocol was reviewed and approved by the Regional and Institutional Ethics Committee at the Clinical Center of the University of Debrecen. Ethical approval number: DE RKEB/IKEB-4843-2017.

### 2.5. Statistical Analysis

The data analysis was carried out using the Statistical Package for Social Sciences (SPSS Inc., Version 28.0, Chicago, IL, USA). Descriptive analysis of overall sitting time was computed for all participants, and separately for sociodemographic and study-related variables. The demographic data are presented as frequencies and percentage (%). The sitting time are presented as means ± standard deviation (SD). To identify subgroups with elevated sitting time, differences between sexes, age groups, BMI index, settlement type, marital status, education, employment status, working hours, work category, sporting habits and physical activity category were determined and mean values of SB (minutes/day sitting) were established. Data normality was evaluated with a Kolgomorov–Smirnov test. A Kruskal–Wallis test and ANOVA analysis were used to analyze the link between sitting time and a rest of variables. Depending on the results of the homogeneity of variances assessed by using Levene statistic at equal variances ANOVA with Tukey post-hoc test was performed; otherwise, a Welch’s test with a Games-Howell post-hoc analysis was carried out. We also calculated the effect size (partial eta squared—η^2^*_p_*—and epsilon-squared—E^2^*_R_*), where η^2^*_p_* ≥ 0.01 is a small, η^2^*_p_* ≥ 0.06 is a medium and η^2^*_p_* ≥ 0.14 is a large effect [58]. Significance level was considered at *p* < 0.05. 

## 3. Results

There were 1295 participants in the study, 47.7% of whom were male, with a mean age of 45.9 years (SD: 15.2). The majority of respondents (58.4%) were married and office workers (55.55%), and 87.9% had completed at least a secondary education. Table 1 summarizes the descriptive data as well as the results of the ANOVA or Welch’s Test. With regard to sex differences, men have a significantly higher sitting time (*p* < 0.001) compared to women with small effect size. The mean level of sitting time differs statistically significantly (*p* < 0.001) for age with a large effect size. Post-hoc analysis revealed a significant difference between the 18–29 age group and the other age groups. This age group reported the highest average sitting time, i.e., 545 min—more than 9 h—and showed the highest prevalence (53%) of sitting at least 8 h per day. With regard to settlement type, residents of the capital have a statistically higher (*p* < 0.001) sitting time with negligible effect size. A significant difference (*p* < 0.001) with a medium effect size was found in the time spent sitting between the different types of work. Post-hoc analysis revealed that sedentary workers spend significantly more time sitting than other workers. An interesting finding is that people in the overweight category by BMI have a significantly (*p* = 0.050) lower average sitting time compared to the other two BMI groups.

In our research, we used both the IPAQ questionnaire and the SBQ questionnaire to ask about the time spent sitting on weekdays and weekend days. Our results show that when asked to estimate the time spent sitting by answering only one question, respondents significantly underestimate the time spent sitting. The average time spent sitting is 288 min (40% of the 12 h during the day) per day during the week and 224 min (31.11% of the day) per day on weekends according to IPAQ. According to the SBQ, this is 469 min per day during the week (65.14% of the daytime period, up by 182 min per or 25.28% compared to the IPAQ) and 421 min per day on the weekend (58.50% of the daytime period, up by 197 min or 27.36% compared to the IPAQ). Thus, there is a difference of more than 25%. This shows that if we have to think about the respondent’s time spent sitting during the day in a list format, we can obtain a more accurate result (Table 2).

The means of the sitting time during working days and weekend days are compared to the characteristics of the population in Table 3. The sex differences in sitting time were statistically significant (*p* < 0.001), with men reporting more sitting time on weekend days: 445 min/day (SD: 243). Especially on working days, declared sitting time decreased with age (*p* < 0.001). Additionally, the proportion of employed people was higher (481 min per day, SD: 263) on working days than unemployed (450 min per day, SD: 248) (*p* = 0.045). As far as the type of employment is concerned, office workers reported 544 min/day (SD: 261) sitting time on working days, while manual workers sat 358 min/day (SD: 221) (*p* < 0.001). 

Table 4 summarizes the difference of various SB between working and weekend days. On weekdays, several specific SBs (listening to music, telephoning, working on computer, sitting during transport) were associated with significantly higher sitting time (*p* < 0.05). On weekends, people tend to spend more time sitting while watching TV, reading and doing arts and crafts (*p* < 0.05). On working days, the average screening time (watching TV, computer games, working on computer) was 286 min, while on the weekends, this was 244 min.

Significant differences by sex were found for almost every sedentary activity on working days (Table 5). Women significantly (*p* < 0.05) watch more TV, work more on computers, read more and do more arts and crafts while sitting on workdays. Men spend a significantly (*p* < 0.05) higher time sitting for a non-work-related computer activity, listening to music, talking on the phone, playing a musical instrument and using public transport during the week. These proportions remain the same on weekends, except for computer work, on which men tend to spend more time on average on weekends.

## 4. Discussions

The present results provide insights into the sedentary lifestyle of Hungarian people through the SBQ and IPAQ questionnaires. In our study, a high level of sitting time was reported; the average daily sitting time on working days was 469 min, while this was 421 min on weekends. In total, 38% of the population sat more than 480 min a day, and 20% of the participants sat more than 600 min a day. Our result suggests that SB is highly prevalent in younger age groups, males, those living in the capital city and those who need to sit for work. We found a relevant difference between the types of measurements in connection with the sitting time. The IPAQ data are significantly lower than data from the SBQ on weekday and weekend days. Consistent with our results, the difference between the two methods has also been reported in a review study [59]. Our study confirmed that more accurate results can be obtained from self-report questionnaires by asking for a list of sedentary activities [59]. 

In Hungary, the activity on weekdays on which the Hungarian adult population spent the most time sitting was working. According to Martínez-Ramos et al. [60], the same can be observed in the USA, Australia, the UK and, according to their own research, in Catalonia (Spain), according to national statistical sources. While in the US [46] and Australia [44,61], the top activity for sitting is watching TV, in Hungary, this is only the case on weekend days. In total, 60% of the US population spent 120 min/day or more sitting and watching television [62]. Belgians spend 128 min/day [41] and the French 120–180 min/day [40] sitting and watching TV. The Hungarian sample reaches this average only on weekend days (130 min/day), while during working days respondents spend less time than this, i.e., 100 min/day on average, sitting watching TV. 

The results of our analysis in a socio-demographic context are similar to those of Martínez-Ramos et al. [60], Bennie et al. [42] and Harrington et al. [48], showing that younger people, sitting workers and more highly educated people have a higher sedentary time. With age, people become more and more inactive, and people’s need for movement decreases. However, it is important to know which physically inactive age groups are at risk and where it is necessary to plan interventions that may increase the level of physical activity and decrease the sitting time. Based on the results of the study, it can be concluded that the group most at risk of sitting inactive lifestyle in terms of age is young adults (18–29 years), due to activities that produce high sedentary time in leisure activities (watching TV, using a phone, using a computer). Proposals aimed at this age group may be important in public health strategies, even with modern ICT tools. Several national [63,64] and international research papers [65] indicated that men tend to be more active in terms of gender compared to women. Our results are in line with those of Matthew et al. [45] and Patel et al. [47], in that men sit more during both working days and on weekend days. Our finding in the Hungarian sample that highly educated men have more sitting time per day is consistent with the findings of Bauman et al. [66] in 20 countries and Chau et al. [43] in Australia. The representativeness of the sample in relation to the settlement type is also important, since studies emphasize the role of village-city and capital settlement types in lifestyle, i.e., activity or inactivity. People living in small settlements, i.e., the village population, move around much more and are more active, which is influenced by the environment [67,68,69,70,71,72,73]. We found similar differences that people living in the capital had higher sitting time, mainly on working days, compared to those living in municipality. Therefore, sitting time spent by this group of the population should be reduced, this requires the introduction of occupational health programs and the offer of extracurricular activities after working hours that are not spent while sitting. This can be seen in well-being strategies affecting the population of the capital, in events that encourage activity, sports parks, adult playgrounds, adventure parks, excursion sites, sports infrastructure and dog-friendly services, which can increase the activity of the capital and provide the population with alternative competitive leisure activities.

Level of education has affected the time spent sitting in such a way that those with higher levels of education sit more on weekdays, presumably because of intellectual work, but the time spent sitting on weekends is higher among those with lower levels of education. The nature of the work also affects inactivity; the seating time of those engaged in intellectual work, and even there of workers who work more than 480 min (8 h) a day, is high, which must be compensated for in workplace health promotion strategies.

In the studies of Golubic [74] and Pulsford [75], overweight people as determined by BMI sit more, but in contrast, in the present study, overweight people have less sitting time than people in the other two BMI categories. In Hungary, more than half (57.3%) of the population has a weight problem and only 42.7% of the population belongs to the health zone. Among the causes of overweight and obesity, lack of movement and inactive lifestyle, SB appears dominantly. This is confirmed by our research, which showed high sitting time during the week in the total sample. It is also shown (Table 3) that those in the obesity category spend significantly more time sitting both during the week and on weekends than those in the overweight category. An interesting result is that those in the normal BMI category have a higher sitting time than those in the overweight category. A possible reason for this result may be that overweight people will try to decrease their sitting time.

In Europe, people’s activity is measured from time to time by the Eurobarometer Sport and physical activity survey. These surveys traditionally ask only one question about the time spent sitting. In 2018, and 2022, 41% and 39% of Europeans, respectively, spent 330 min (5.5 h) or more sitting each day. In these two studies, 12% (2018) and 11% (2022) of the European population spent more than 510 min (8.5 h) sitting per day. A list of activities spent sitting would raise this already high figure even higher. It would be important to confront society as much as possible with the issue of time spent sitting as a matter of importance. Today’s highly sedentary lifestyles suggest that individuals spend significantly more than 360 min (6 h) on a single SB [76]. In the present study, the average daily sedentary time for the Hungarian population exceeds 420 min (7 h). Hungary has one of the highest obesity rates in Europe. Although 85% of Hungarian adults reached the recommended physical activity levels in 2009 according to the Hungarian Central Statistical Office [77] and 35% of adults (18–64 years) had a sufficient physical activity level in 2021 according to Eurostat [78], physical activity cannot fully compensate the negative health effect derived from the large amount of prolonged sitting time. This is especially true among young people and office workers.

The negative effects of the COVID-19 pandemic on SB, especially on sitting time is alarming, as proved by several studies [52,79,80,81,82,83,84,85,86,87,88,89,90,91]. During this period, the sitting time significantly increased, which was even worsened the already high amount of sitting time. This period has delivered an extremely important message for the sedentary people: avoid high level of SB and uninterrupted sitting.

There are a number of recommendations [92,93,94,95] that specify the amount of weekly or daily physical activity recommended for different age groups to offset the adverse health effects of a sedentary lifestyle through physical activity or recreational exercise. A number of studies have reported that giving up a sedentary lifestyle or reducing the amount of time spent in sedentary activities and increasing physical activity leads to positive health changes. Gregg [96] found that women who were physically active at baseline and 6 years later had a 32% lower incidence of coronary heart disease and a 38% lower mortality from coronary heart disease than women who were sedentary at both time points in the survey. However, Chomistek et al. [97] has shown that prolonged sedentary time is associated with a risk of cardiovascular disease, irrespective of leisure-time physical activity, i.e., the health risk of sitting for more than 600 min (10 h) per day cannot be fully compensated for by physical activity. 

### Limitations

One of the main limitations of our study is the self-reported data. However, the most popular and cost-effective methods to give a general estimation of SB and sedentary time are still self-report questionnaires. Accelerometers have been used mainly in smaller randomized controlled trials. In large studies, self-report questionnaires are preferred because of the high cost of the technology. Several studies have compared the validity of the different measurement types and found that questionnaires underestimate sitting time [50,59,98,99,100]. In a review by Prince et al. [59], the average mean difference was −105.19 min/day (95% CI: −127.21, −83.17); self-report underestimated sedentary time by 104.4 min/day compared to device measures. Sansano-Nadal et al. [101] showed a 73 min/day mean difference between the two measures, while Kastelic and Šarabon [76] revealed greater mean bias (181 min/day). Another limitation of present research is the SBQ measurement scale itself because it already reveals the underestimation of the sedentary time, particularly the maximum category of ≥360 min (6 h). This characteristic of the scale may lead to underestimation. However, self-report measures tended to be more reliable in assessing different sedentary activities to obtain domain-specific information [12]. In addition, self-reported data could be biased by false information due to social desirability of the participants. Furthermore, the time spent sitting can be distorted by the fact that sedentary activities are performed in parallel. Self-reported weight and height data can be considered as a limitation in our research. Another limitation of the present study is derived from the cross-sectional study design, which cannot be used to analyze behavior over a period to time and does not help determine a cause and effect relationship. The well-known drawbacks of online survey data collection can also be a limitation of this study. These limitations should be considered in future studies.

## 5. Conclusions

The present study gives first insights into the overall sitting time for the Hungarian adult population. The present study may also contribute to the debate on the self-report measures of SB. Bearing in mind the context of the development of and widespread familiarity with objective measurement methods, self-report and multi-item questionnaires are still the most widely used methods to assess SB. Our results clearly show that the self-report single item measure (IPAQ) significantly underestimates sedentary time compared to the multi-item questionnaire (SBQ). In addition to this, self-reported measures generally have poor accuracy and underestimate the time spent in SB. Therefore, future research should consider to use mixed methodology (subjective and objective) to measure sitting time and capture important domain specific sitting time information on weekdays and weekend days [102].

As far as the negative health effects of sitting time and especially prolonged sitting time are concerned, which is an emerging public health problem, SB should be measured in population research instead of being defined by a lack of physical activity [37]. Our alarming results may draw attention to the fact that active lifestyles are explicitly needed to reduce inactivity-related and prolonged sitting-related negative health consequences and their costs. However, in addition to increasing physical activity, it is also important to reduce effective sitting time, as it may reach a level that can no longer be compensated for by physical activity and sport. Identifying vulnerable groups with high sitting times is very important and should be taken into account in public health strategies. In Hungary, but also in OECD countries, cardiovascular diseases are the leading cause of morbidity and mortality, and one of the main causes of these diseases is a sedentary lifestyle. The nutritional status of Hungarians is unfavorable and more than half of the population struggling with weight problems, which also increases the risk of NCDs. Consequently, adult population should be continuously surveyed, and requires specific interventions and strategies that particularly counteract the increased sitting time, especially among vulnerable groups.

## Figures and Tables

**Table 1:** Characteristics of the sample and analysis of the variance for daily sitting time.

		*N*	%	Average Daily Sitting Time (min)	SD	*p* Value and (η^2^*_p_* or E^2^_R_ Value)	Sitting Time < 480 min *N* (%)	Sitting Time > 480 min *N* (%)
Sex	Man	618	47.7	470.25	236.39	<0.001 *	370 (59.9)	248 (40.1)
	Woman	677	52.3	443.19	238.82	(0.003)	432 (63.8)	245 (36.2)
Age group (years)	18–29	251	19.4	545.12	301.90	<0.001 *	118 (47)	133 (53)
	30–39	239	18.5	457.35	235.17	(0.370)	148 (61.9)	91 (38.1)
	40–49	267	20.6	425.53	226.96		179 (67)	88 (33)
	50–59	208	16.1	445.95	208.63		138 (66.3)	70 (33.7)
	60+	330	25.4	419.52	191.47		218 (66.1)	112 (33.9)
Education level	Maximum primary school Secondary, high school education	757	58.5	461.32	252.49	0.395	466 (61.6)	291 (38.4)
	College/University, degree	538	41.5	450.11	230.32	(0.007)	335 (62.3)	203 (37.7)
Settlement type	Capital city	224	17.3	509.08	247.60	<0.001 *	119 (53.1)	105 (46.9)
	County town	327	25.3	460.34	228.19	(0.012)	202 (61.8)	125 (38.2)
	City	484	37.4	440.17	229.61		311 (64.3)	173 (35.7)
	Municipality	260	20.1	434.87	250.66		169 (65)	91 (35)
Employment status	Employed	822	63.5	463.92	236.12	0.120	498 (60.6)	324 (39.4)
	Unemployed	473	36.5	442.48	240.76	(0.002)	303 (64.2)	169 (35.8)
Work category	Sitting Work (Office)	614	47.4	514.82	241.28	<0.001 *	311 (50.7)	303 (49.3)
	Mixed work (sitting-standing, e.g., teacher, dentist)	386	29.8	430.90	228.63	(0.064)	264 (68.4)	122 (31.6)
	Standing work (shop assistant, hairdresser, etc.)	102	7.9	360.22	192.76		81 (79.4)	21 (20.6)
	Physical work (electricians, furniture, etc.) Heavy physical work (masonry, miner, construction worker, etc.)	193	14.9	370.93	216.67		145 (75.1)	48 (24.9)
Working hours	480 min (8 h)	737	56.9	454.95	231.61	0.735	448 (60.8)	289 (39.2)
	Less than 480 min (8 h, part-time employment)	355	27.4	447.88	233.93	(0.001)	223 (62.6)	133 (37.4)
	More than 480 min (8 h, frequent overtime)	190	14.7	468.97	267.39		126 (66.3)	64 (33.7)
Marital status	Married	756	58.4	435.63	230.54	<0.001 *	500 (66.1)	256 (33.9)
	Single, Divorced, Widower	539	41.6	484.78	245.35	(0.010)	302 (56)	238 (44)
BMI (kg/m^2^)	Normal weight (18.50–24.99)	523	40.4	469.14	251.72	0.050 *	315 (60.2)	208 (39.8)
	Overweight (25.00–29.99)	436	33.7	433.69	22.38	(0.006)	287 (65.8)	149 (34.2)
	Obesity (>30.00)	306	23.6	469.81	232.25		176 (57.5)	130 (42.5)
Sport activity	Yes	532	41.1	465.06	245.20	0.271	325 (61.1)	207 (38.9)
	No	762	58.9	449.80	232.72	(0.001)	476 (62.5)	286 (37.5)

SD = Standard Deviation, η^2^*_p_* = partial eta squared, E^2^*_R_*= epsilon-squared. * *p* < 0.05 for the difference.

**Table 2:** Sitting time difference between IPAQ and SBQ measurement.

	IPAQ		SBQ	
Days	Working Days	Weekend Days	Working Days	Weekend Days
Mean (min)	287.82	224.30	469.53	421.25
N	1153	1117	1292	1287
SD	233.37	179.96	258.10	241.72

SD = Standard Deviation; Mean (min) = Average sitting time in minutes.

**Table 3:** Mean sitting time (SBQ) in minutes with respect to the different categories of the sample in weekdays and weekend days.

		Working Days			Weekend Days		
		Mean	SD	*p* Value	Mean	SD	*p* Value
Total		469.53	258.09		421.25	241.41	
Sex	Man	479.29	257.03	0.195	444.95	242.91	<0.001 *
	Woman	460.23	258.94		399.71	238.77	
Age group (years)	18–29	551.04	321.79	<0.001 *	526.35	312.89	<0.001 *
	30–39	475.47	258.29		408.22	235.81	
	40–49	440.26	251.26		388.69	226.47	
	50–59	460.67	235.42		409.16	206.15	
	60+	433.02	205.62		385.77	191.71	
Educational level	Maximum primary school Secondary, high school education	470.63	271.50	0.870	437.90	256.03	0.007 *
	College/University, degree	468.29	242.28		402.32	223.04	
	Capital city	531.09	267.65	<0.001 *	454.04	25.72	0.172
Settlement type	County town	478.12	245.76		415.54	240.42	
	City	450.21	249.44		412.46	232.53	
	Municipality	442.25	272.50		416.60	243.96	
	Employed	480.89	262.98	0.045 *	420.18	233.97	0.834
Employment status	Unemployed	449.76	248.40		423.11	254.84	
	Sitting Work (Office)	543.93	261.32	<0.001 *	440.08	251.79	0.015 *
Work category	Mixed work (sitting-standing, e.g.: teacher, dentist)	436.45	246.04		415.07	22.22	
	Standing work (shop assistant, hairdresser, etc.)	358.22	202.78		365.24	210.90	
	Physical work (electricians, furniture, etc.) Heavy physical work (masonry, miner, construction worker, etc.)	357.84	221.07		403.64	243.71	
Working hours	480 min (8 h)	471.68	254.49	0.659	411.00	235.11	0.440
	Less than 480 min (8 h, part-time employment)	457.65	246.62		423.46	239.12	
	More than 480 min (8 h, frequent overtime)	477.01	290.78		448.88	269.26	
	Married	451.07	250.75	0.002 *	396.00	228.29	<0.001 *
Marital status	Single, Divorced, Widower	495.41	266.14		456.70	255.43	
	Normal weight (18.50–24.99)	481.25	271.30	0.112	426.42	259.20	0.007 *
BMI (kg/m^2^)	Overweight (25.00–29.99)	448.51	246.00		395.63	216.20	
	Obesity (>30.00)	478.37	249.28		448.43	240.63	
Sport activity	Yes	480.35	262.79	0.210	425.03	253.81	0.645
	No	461.96	254.67		418.61	233.02	
Moving categories	Low	534.08	328.83	<0.001 *	487.91	313.90	0.018 *
	Moderate	494.32	237.83		407.53	215.50	
	High	448.14	248.53		414.68	234.93	

SD = Standard deviation; Mean = Average sitting time in minutes. * *p* < 0.05 for the difference.

**Table 4:** Difference between days with respect to time spent sitting per sedentary behavior in the sample.

Item	Working Day					Weekend Day					
	Mean (min)	SD	Median	Percentile 25	Percentile 75	Mean (min)	SD	Median	Percentile 25	Percentile 75	*p*-Value
Television	100.13	80.75	60	30	120	130.28	93.6	120	60	180	<0.001 *
Computer (no work)	59.04	82.52	30	0	60	59.81	87.39	15	0	60	0.629
Sit listen to music	43	73.65	15	0	60	39.48	66.92	15	0	60	0.040 *
Sit talk on telephone	28.49	44.98	15	15	30	22.14	32.61	15	0	30	<0.001 *
Computer (work)	126.47	130.57	60	15	240	54.23	73.65	30	0	60	<0.001 *
Reading (no computer)	44.75	52.79	30	15	60	53	60.35	30	15	60	<0.001 *
Play musical instrument	7.05	32.77	0	0	0	8.39	36	0	0	0	0.081
Arts and crafts	12.1	40.12	0	0	0	14.4	41.45	0	0	0	0.001 *
Sitting during transport	50.62	63.65	30	15	60	37.95	52.91	30	0	60	<0.001 *

SD = Standard deviation; Mean (min). = Average sitting time in minutes. * *p* < 0.05 for the difference.

**Table 5:** Difference between sexes with respect to time spent sitting per sedentary occupation.

Item		Working Day				Weekend Day			
	Sex	*N*	Mean	SD	*p* Value	*N*	Mean	SD	*p* Value
Television	Man	618	94.48	78.18	<0.001 *	618	126.44	93.09	0.158
	Woman	677	105.29	82.74		677	133.79	94.00	
Computer (no work)	Man	618	66.91	88.25	<0.001 *	618	71.47	96.07	<0.001 *
	Woman	677	51.85	76.27		677	49.16	77.18	
Sit listen to music	Man	618	51.37	77.34	<0.001 *	618	49.62	75.21	<0.001 *
	Woman	677	35.35	69.29		677	30.22	56.83	
Sit talk on telephone	Man	618	29.74	43.35	<0.001 *	618	22.69	31.93	0.564
	Woman	677	27.35	46.42		677	21.64	33.24	
Computer (work)	Man	618	118.73	125.08	0.339	618	58.17	75.30	0.066
	Woman	677	133.54	135.10		677	50.63	71.98	
Reading (no computer)	Man	618	40.27	48.90	<0.001 *	618	49.42	58.07	<0.001 *
	Woman	677	48.83	55.83		677	56.26	62.22	
Play musical instrument	Man	618	9.00	33.55	<0.001 *	618	12.28	45.03	<0.001 *
	Woman	677	5.27	31.96		677	4.83	24.57	
Arts and crafts	Man	618	8.12	31.36	<0.001 *	618	10.39	35.72	<0.001 *
	Woman	677	15.74	46.42		677	18.05	45.79	
Sitting during transport	Man	618	60.22	68.73	<0.001 *	618	42.77	53.58	<0.001 *
	Woman	677	41.87	57.30		677	33.55	51.94	

SD = Standard deviation; Mean (min). = Average sitting time in minutes. * *p* < 0.05 for the difference.

## Data Availability

The datasets generated for this study are available on request to the corresponding author.

## References

[1] Dunstan D.W., Healy G.N., Sugiyama T., Owen N. (2010). Too Much Sitting and Metabolic Risk—Has Modern Technology Caught Up with Us?. Eur. Endocrinol..

[2] Owen N., Healy G.N., Matthews C.E., Dunstan D.W. (2010). Too Much Sitting: The Population Health Science of Sedentary Behavior. Exerc. Sport Sci. Rev..

[3] Salmon J., Tremblay M.S., Marshall S.J., Hume C. (2011). Health Risks, Correlates, and Interventions to Reduce Sedentary Behavior in Young People. Am. J. Prev. Med..

[4] van Ekris E., Altenburg T.M., Singh A.S., Proper K.I., Heymans M.W., Chinapaw M.J.M. (2016). An Evidence-Update on the Prospective Relationship between Childhood Sedentary Behaviour and Biomedical Health Indicators: A Systematic Review and Meta-Analysis. Obes. Rev..

[5] Poses-Ferrer E., Parisi R., Gonzalez-Viana A., Castell C., Arias de la Torre J., Jones A., Serra-Sutton V., Espallargues M., Cabezas C. (2022). Erratum to: Daily Sitting Time and its Association with Non-Communicable Diseases and Multimorbidity in Catalonia. Eur. J. Public Health.

[6] Biswas A., Oh P.I., Faulkner G.E., Bajaj R.R., Silver M.A., Mitchell M.S., Alter D.A. (2015). Sedentary Time and its Association with Risk for Disease Incidence, Mortality, and Hospitalization in Adults a Systematic Review and Meta-Analysis. Ann. Intern. Med..

[7] Ford E.S., Caspersen C.J. (2012). Sedentary Behaviour and Cardiovascular Disease: A Review of Prospective Studies. Int. J. Epidemiol..

[8] Young D., Hivert M., Alhassan S., Camhi S., Ferguson J., Katzmarzyk P., Lewis C., Owen N., Perry C., Siddique J. (2016). Sedentary Behavior and Cardiovascular Morbidity and Mortality: A Science Advisory from the American Heart Association. Circulation.

[9] Chen D., Wu H., Wang X., Huang T., Jia J. (2022). Shared Genetic Basis and Causal Relationship between Television Watching, Breakfast Skipping and Type 2 Diabetes: Evidence from a Comprehensive Genetic Analysis. Front. Endocrinol..

[10] Chau J.Y., Grunseit A.C., Chey T., Stamatakis E., Brown W.J., Matthews C.E., Bauman A.E., van der Ploeg H.P. (2013). Daily Sitting Time and all-Cause Mortality: A Meta-Analysis. PLoS ONE.

[11] Ekelund U., Steene-Johannessen J., Brown W.J., Fagerland M.W., Owen N., Powell K.E., Bauman A., Lee I. (2016). Does Physical Activity Attenuate, Or Even Eliminate, the Detrimental Association of Sitting Time with Mortality? A Harmonised Meta-Analysis of Data from More than 1 Million Men and Women. Lancet.

[12] Henschel B., Gorczyca A.M., Chomistek A.K. (2020). Time Spent Sitting as an Independent Risk Factor for Cardiovascular Disease. Am. J. Lifestyle Med..

[13] World Health Organization WHO (2016). Physical Activity Strategy for the WHO European Region 2016–2025. https://apps.who.int/iris/handle/10665/329407.

[14] Koedijk J.B., van Rijswijk J., Oranje W.A., van den Bergh J.P., Bours S.P., Savelberg H.H., Schaper N.C. (2017). Sedentary Behaviour and Bone Health in Children, Adolescents and Young Adults: A Systematic Review. Osteoporos Int..

[15] Bort-Roig J., Briones-Buixassa L., Felez-Nobrega M., Guàrdia-Sancho A., Sitjà-Rabert M., Puig-Ribera A. (2020). Sedentary Behaviour Associations with Health Outcomes in People with Severe Mental Illness: A Systematic Review. Eur. J. Public Health.

[16] Biddle S.J.H., Bengoechea García E., Pedisic Z., Bennie J., Vergeer I., Wiesner G. (2017). Screen Time, Other Sedentary Behaviours, and Obesity Risk in Adults: A Review of Reviews. Curr. Obes. Rep..

[17] Güngör N.K. (2014). Overweight and Obesity in Children and Adolescents. Clin. Res. Pediatr. Endocrinol..

[18] Lee I., Shiroma E.J., Lobelo F., Puska P., Blair S.N., Katzmarzyk P.T. (2012). Effect of Physical Inactivity on Major Non-Communicable Diseases Worldwide: An Analysis of Burden of Disease and Life Expectancy. Lancet.

[19] Strain T., Brage S., Sharp S.J., Richards J., Tainio M., Ding D., Benichou J., Kelly P. (2020). Use of the Prevented Fraction for the Population to Determine Deaths Averted by Existing Prevalence of Physical Activity: A Descriptive Study. Lancet Glob. Health.

[20] WHO/Europe (2022). Noncommunicable Diseases. Key Facts. https://www.who.int/news-room/fact-sheets/detail/noncommunicable-diseases.

[21] Matusiak-Wieczorek E., Lipert A., Kochan E., Jegier A. (2020). The Time Spent Sitting does Not always Mean a Low Level of Physical Activity. BMC Public Health.

[22] Tremblay M.S., Aubert S., Barnes J.D., Saunders T.J., Carson V., Latimer-Cheung A.E., Chastin S.F.M., Altenburg T.M., Chinapaw M.J.M., Aminian S. (2017). Sedentary Behavior Research Network (SBRN)—Terminology Consensus Project Process and Outcome. Int. J. Behav. Nutr. Phys. Act..

[23] van der Ploeg H.P., Hillsdon M. (2017). Is Sedentary Behaviour just Physical Inactivity by another Name?. Int. J. Behav. Nutr. Phy..

[24] (2018). World Health Organization Hungary Physical Activity Factsheet 2018. https://www.euro.who.int/__data/assets/pdf_file/0003/288111/HUNGARY-Physical-Activity-Factsheet.pdf.

[25] Owen N., Sugiyama T., Eakin E.E., Gardiner P.A., Tremblay M.S., Sallis J.F. (2011). Adults’ Sedentary Behavior Determinants and Interventions. Am. J. Prev. Med..

[26] Wang N.X., Chen J., Wagner N.L., Rebello S.A., Petrunoff N.A., Owen N., Mueller-Riemenschneider F. (2020). Understanding and Influencing Occupational Sedentary Behavior: A Mixed-Methods Approach in a Multiethnic Asian Population. Health Educ. Behav..

[27] Ryan C.G., Dall P.M., Granat M.H., Grant P.M. (2011). Sitting Patterns at Work: Objective Measurement of Adherence to Current Recommendations. Ergonomics.

[28] Kazi A., Duncan M., Clemes S., Haslam C. (2014). A Survey of Sitting Time among UK Employees. Occup. Med-Oxf..

[29] Ráthonyi G., Kósa K., Bács Z., Ráthonyi-Ódor K., Füzesi I., Lengyel P., Bácsné Bába É. (2021). Changes in Workers’ Physical Activity and Sedentary Behavior during the COVID-19 Pandemic. Sustainability.

[30] Martinez-Gonzalez M.A., Martinez J.A., Hu F.B., Gibney M.J., Kearney J. (1999). Physical Inactivity, Sedentary Lifestyle and Obesity in the European Union. Int. J. Obes..

[31] Salmon J., Bauman A., Crawford D., Timperio A., Owen N. (2000). Association between Television Viewing and Overweight among Australian Adults Participating in Varying Levels of Leisure-Time Physical Activity. Int. J. Obes..

[32] Hu F.B., Leitzmann M.F., Stampfer M.J., Colditz G.A., Willett W.C., Rimm E.B. (2001). Physical Activity and Television Watching in Relation to Risk for Type 2 Diabetes Mellitus in Men. Arch. Intern. Med..

[33] Hu F.B., Li T.Y., Colditz G.A., Willett W.C., Manson J.E. (2003). Television Watching and Other Sedentary Behaviors in Relation to Risk of Obesity and Type 2 Diabetes Mellitus in Women. J. Am. Med. Assoc..

[34] Hamilton M.T., Hamilton D.G., Zderic T.W. (2007). Role of Low Energy Expenditure and Sitting in Obesity, Metabolic Syndrome, Type 2 Diabetes, and Cardiovascular Disease. Diabetes.

[35] Chastin S.F.M., Granat M.H. (2009). Methods for Objective Measure, Quantification and Analysis of Sedentary Behaviour and Inactivity. Gait Posture.

[36] Prince S.A., LeBlanc A.G., Colley R.C., Saunders T.J. (2017). Measurement of Sedentary Behaviour in Population Health Surveys: A Review and Recommendations. Peer J..

[37] Rosenberg D.E., Norman G.J., Wagner N., Patrick K., Calfas K.J., Sallis J.F. (2010). Reliability and Validity of the Sedentary Behavior Questionnaire (SBQ) for Adults. J. Phys. Act. Health.

[38] Aunger J., Wagnild J. (2022). Objective and Subjective Measurement of Sedentary Behavior in Human Adults: A Toolkit. Am. J. Hum. Biol..

[39] Healy G.N., Dunstan D.W., Salmon J., Cerin E., Shaw J.E., Zimmet P.Z., Owen N. (2007). Objectively Measured Light-Intensity Physical Activity is Independently Associated with 2-H Plasma Glucose. Diabetes Care.

[40] Bertrais S., Preziosi P., Mennen L., Galan P., Hercberg S., Oppert J. (2004). Sociodemographic and Geographic Correlates of Meeting Current Recommendations for Physical Activity in Middle-Aged French Adults: The Supplementation En Vitamines Et Mineraux Antioxydants (SUVIMAX) Study. Am. J. Public Health.

[41] Van Dyck D., Cardon G., Deforche B., Owen N., De Cocker K., Wijndaele K., De Bourdeaudhuij I. (2011). Socio-Demographic, Psychosocial and Home-Environmental Attributes Associated with Adults’ Domestic Screen Time. BMC Public Health.

[42] Bennie J.A., Chau J.Y., van der Ploeg H.P., Stamatakis E., Do A., Bauman A. (2013). Prevalence and Correlates of Sitting in European Adults—A Comparison of 32 Eurobarometer-Participating Countries. Int. J. Behav. Nutr. Phys..

[43] Chau J.Y., Merom D., Grunseit A., Rissel C., Bauman A.E., van der Ploeg H.P. (2012). Temporal Trends in Non-Occupational Sedentary Behaviours from Australian Time use Surveys 1992, 1997 and 2006. Int. J. Behav. Nutr. Phys..

[44] Sugiyama T., Salmon J., Dunstan D.W., Bauman A.E., Owen N. (2007). Neighborhood Walkability and TV Viewing Time among Australian Adults. Am. J. Prev. Med..

[45] Matthews C.E., Chen K.Y., Freedson P.S., Buchowski M.S., Beech B.M., Pate R.R., Troiano R.P. (2008). Amount of Time Spent in Sedentary Behaviors in the United States, 2003–2004. Am. J. Epidemiol..

[46] King A.C., Goldberg J.H., Salmon J., Owen N., Dunstan D., Weber D., Doyle C., Robinson T.N. (2010). Identifying Subgroups of U.S. Adults at Risk for Prolonged Television Viewing to Inform Program Development. Am. J. Prev. Med..

[47] Patel A.V., Bernstein L., Deka A., Feigelson H.S., Campbell P.T., Gapstur S.M., Colditz G.A., Thun M.J. (2010). Leisure Time Spent Sitting in Relation to Total Mortality in a Prospective Cohort of US Adults. Am. J. Epidemiol..

[48] Harrington D.M., Barreira T.V., Staiano A.E., Katzmarzyk P.T. (2013). The Descriptive Epidemiology of Sitting among US Adults, NHANES 2009/2010. J. Sci. Med. Sport.

[49] Bakar Y., Tugral A., Ozel A., Devran Altuntas Y., Yakut Y. (2018). Reliability and Validity of Sedentary Behavior Questionnaire in Turkish Population: Evaluation of Psychometric Properties. Middle East J. Rehabil. Health Stud..

[50] Chastin S.F.M., Dontje M.L., Skelton D.A., Čukić I., Shaw R.J., Gill J.M.R., Greig C.A., Gale C.R., Deary I.J., Der G. (2018). Systematic Comparative Validation of Self-Report Measures of Sedentary Time Against an Objective Measure of Postural Sitting (activPAL). Int. J. Behav. Nutr. Phys..

[51] Bakker E.A., Hopman M.T.E., Lee D., Verbeek A.L.M., Thijssen D.H.J., Eijsvogels T.M.H. (2020). Correlates of Total and Domain-Specific Sedentary Behavior: A Cross-Sectional Study in Dutch Adults. BMC Public Health.

[52] Dor-Haim H., Katzburg S., Revach P., Levine H., Barak S. (2021). The Impact of COVID-19 Lockdown on Physical Activity and Weight Gain among Active Adult Population in Israel: A Cross-Sectional Study. BMC Public Health.

[53] Prince S.A., Reid R.D., Bernick J., Clarke A.E., Reed J.L. (2018). Single versus multi-item self-assessment of sedentary behaviour: A comparison with objectively measured sedentary time in nurses. J. Sci. Med. Sport.

[54] Bácsné Bába É., Ráthonyi G., Müller A., Ráthonyi-Odor K., Balogh P., Ádány R., Bács Z. (2020). Physical Activity of the Population of the most Obese Country in Europe, Hungary. Front. Public Health.

[55] (2022). The International Physical Activity Questionnaire. https://sites.google.com/site/theipaq/.

[56] Patterson R., McNamara E., Tainio M., de Sá T.H., Smith A.D., Sharp S.J., Edwards P., Woodcock J., Brage S., Wijndaele K. (2018). Sedentary Behaviour and Risk of all-Cause, Cardiovascular and Cancer Mortality, and Incident Type 2 Diabetes: A Systematic Review and Dose Response Meta-Analysis. Eur. J. Epidemiol..

[57] Vélez Álvarez C., Vidarte Claros J.A., Arango Arenas A., Patiño Palma B.E., Rondón Villamil Y.A. (2021). Adaptation and Validation of Content of the Sedentary Behavior Questionnaire. Hacia La Promocíon De La Salud.

[58] Cohen J. (1988). Statistical Power Analysis for the Behavioral Sciences.

[59] Prince S.A., Cardilli L., Reed J.L., Saunders T.J., Kite C., Douillette K., Fournier K., Buckley J.P. (2020). A Comparison of Self-Reported and Device Measured Sedentary Behaviour in Adults: A Systematic Review and Meta-Analysis. Int. J. Behav. Nutr. Phys..

[60] Martínez-Ramos E., Beltran A., Martín-Borràs C., Lasaosa-Medina L., Real J., Trujillo J., Solà-Gonfaus M., Puigdomenech E., Castillo-Ramos E., Puig-Ribera A. (2018). Patterns of Sedentary Behavior in Overweight and Moderately Obese Users of the Catalan Primary-Health Care System. PLoS ONE.

[61] Vandelanotte C., Sugiyama T., Gardiner P., Owen N. (2009). Associations of Leisure-Time Internet and Computer use with Overweight and Obesity, Physical Activity and Sedentary Behaviors: Cross-Sectional Study. J. Med. Internet. Res..

[62] Yang L., Cao C., Kantor E.D., Nguyen L.H., Zheng X., Park Y., Giovannucci E.L., Matthews C.E., Colditz G.A., Cao Y. (2019). Trends in Sedentary Behavior among the US Population, 2001—2016. J. Am. Med. Assoc..

[63] Győri F., Berki T., Katona Z., Vári B., Katona Z., Petrovszki Z. (2021). Physical Activity in the Southern Great Plain Region of Hungary: The Role of Sociodemographics and Body Mass Index. Int. J. Environ. Res. Public Health.

[64] Makai A., Füge K., Breitenbach Z., Figler M. (2016). Physical Activity Levels of Adults in Two Counties of Hungary in View of Sociodemographic Inequalities. Health Probl. Civiliz..

[65] (2022). European Commission, Directorate-General for Education, Youth, Sport and Culture, Sport and Physical Activity: Full Report. Publications Office of the European Union. https://op.europa.eu/en/publication-detail/-/publication/c601d8fb-3e0d-11ed-92ed-01aa75ed71a1/language-en.

[66] Bauman A., Ainsworth B.E., Sallis J.F., Hagströmer M., Craig C.L., Bull F.C., Pratt M., Venugopal K., Chau J., Sjöström M. (2011). The Descriptive Epidemiology of Sitting. A 20-Country Comparison using the International Physical Activity Questionnaire (IPAQ). Am. J. Prev. Med..

[67] Loucaides C.A., Chedzoy S.M., Bennett N. (2004). Differences in Physical Activity Levels between Urban and Rural School Children in Cyprus. Health Educ. Res..

[68] Yamauchi T., Umezaki M., Ohtsuka R. (2001). Influence of Urbanisation on Physical Activity and Dietary Changes in Huli-Speaking Population: A Comparative Study of Village Dwellers and Migrants in Urban Settlements. Br. J. Nutr..

[69] Sigmundova D., Sigmund E., Froemel K., Vlkova P. (2009). Pilot Study on the Application of the Nqls Questionnaire in a Study of Physical Activity in Inhabitants of Villages and Towns. Acta Univ. Palacki. Olomuc. Gymn..

[70] Urbina Casasola Y. (2014). Espacio Público Urbano Como Catalizador De Actividad Física Y Bienestar Psicológico. Rev. Wímb Lu..

[71] Christian H.E., Bull F.C., Middleton N.J., Knuiman M.W., Divitini M.L., Hooper P., Amarasinghe A., Giles-Corti B. (2011). How Important is the Land use Mix Measure in Understanding Walking Behaviour? Results from the RESIDE Study. Int. J. Behav. Nutr. Phys. Act..

[72] Dennis M., James P. (2017). Evaluating the Relative Influence on Population Health of Domestic Gardens and Green Space Along a Rural-Urban Gradient. Landsc. Urban Plan..

[73] Belanche D., Casaló L.V., Orús C. (2016). City Attachment and use of Urban Services: Benefits for Smart Cities. Cities.

[74] Golubic R., Wijndaele K., Sharp S.J., Simmons R.K., Griffin S.J., Wareham N.J., Ekelund U., Brage S. (2015). Physical Activity, Sedentary Time and Gain in overall and Central Body Fat: 7-Year Follow-Up of the ProActive Trial Cohort. Int. J. Obes..

[75] Pulsford R.M., Stamatakis E., Britton A.R., Brunner E.J., Hillsdon M.M. (2013). Sitting Behavior and Obesity: Evidence from the Whitehall II Study. Am. J. Prev. Med..

[76] Kastelic K., Šarabon N. (2019). Comparison of Self-Reported Sedentary Time on Weekdays with an Objective Measure (activPAL). Meas. Phys. Educ. Exerc. Sci..

[77] Központi Statisztikai Hivatal Európai Lakossági Egészségfelmérés. Budapest. Magyarország.. https://www.ksh.hu/docs/hun/xftp/idoszaki/elef/elef_2009_osszefoglalo.pdf.

[78] European Commission Hungary Physical Activity Factsheet 2021. https://sport.ec.europa.eu/document/hungary-physical-activity-factsheet-2021.

[79] Zheng C., Huang W.Y., Sheridan S., Sit C.H., Chen X., Wong S.H. (2020). COVID-19 Pandemic Brings a Sedentary Lifestyle in Young Adults: A Cross-Sectional and Longitudinal Study. Int. J. Environ. Res. Public Health.

[80] Romero-Blanco C., Rodríguez-Almagro J., Onieva-Zafra M.D., Parra-Fernández M.L., Prado-Laguna M.D.C., Hernández-Martínez A. (2020). Physical Activity and Sedentary Lifestyle in University Students: Changes during Confinement due to the COVID-19 Pandemic. Int. J. Environ. Res. Public Health.

[81] Bentlage E., Ammar A., How D., Ahmed M., Trabelsi K., Chtourou H., Brach M. (2020). Practical Recommendations for Maintaining Active Lifestyle during the COVID-19 Pandemic: A Systematic Literature Review. Int. J. Environ. Res. Public Health.

[82] Hermassi S., Hayes L.D., Salman A., Sanal-Hayes N.E.M., Abassi E., Al-Kuwari L., Aldous N., Musa N., Alyafei A., Bouhafs E.G. (2021). Physical Activity, Sedentary Behavior, and Satisfaction with Life of University Students in Qatar: Changes during Confinement due to the COVID-19 Pandemic. Front. Physiol..

[83] Jalloun R.A., Alahmadi M.A. (2022). The Impact of COVID-19 Crises on the Diet Quality, Physical Activity, and Sedentary Lifestyle among Saudi’s Adults COVID-19 Crises on the Diet Quality, Physical Activity, and Sedentary Lifestyle. Prog. Nutr..

[84] Musa S., Elyamani R., Dergaa I. (2022). COVID-19 and Screen-Based Sedentary Behaviour: Systematic Review of Digital Screen Time and Metabolic Syndrome in Adolescents. PLoS ONE.

[85] Kharel M., Sakamoto J.L., Carandang R.R., Ulambayar S., Shibanuma A., Yarotskaya E., Basargina M., Jimba M. (2022). Impact of COVID-19 Pandemic Lockdown on Movement Behaviours of Children and Adolescents: A Systematic Review. BMJ Glob. Health.

[86] Sañudo B., Fennell C., Sánchez-Oliver A.J. (2020). Objectively-Assessed Physical Activity, Sedentary Behavior, Smartphone use, and Sleep Patterns Pre- and during-COVID-19 Quarantine in Young Adults from Spain. Sustainability.

[87] Castañeda-Babarro A., Arbillaga-Etxarri A., Gutiérrez-Santamaría B., Coca A. (2020). Physical Activity Change during COVID-19 Confinement. Int. J. Environ. Res. Public Health.

[88] Cheval B., Sivaramakrishnan H., Maltagliati S., Fessler L., Forestier C., Sarrazin P., Orsholits D., Chalabaev A., Sander D., Ntoumanis N. (2021). Relationships between Changes in Self-Reported Physical Activity, Sedentary Behaviour and Health during the Coronavirus (COVID-19) Pandemic in France and Switzerland. J. Sport Sci..

[89] Stockwell S., Trott M., Tully M., Shin J., Barnett Y., Butler L., McDermott D., Schuch F., Smith L. (2021). Changes in Physical Activity and Sedentary Behaviours from before to during the COVID-19 Pandemic Lockdown: A Systematic Review. BMJ Open Sport Exerc. Med..

[90] Bertrand L., Shaw K.A., Ko J., Deprez D., Chilibeck P.D., Zello G.A. (2021). The Impact of the Coronavirus Disease 2019 (COVID-19) Pandemic on University Students’ Dietary Intake, Physical Activity, and Sedentary Behaviour. Appl. Physiol. Nutr. Metab..

[91] Luciano F., Cenacchi V., Vegro V., Pavei G. (2021). COVID-19 Lockdown: Physical Activity, Sedentary Behaviour and Sleep in Italian Medicine Students. Eur. J. Sport Sci..

[92] Special Eurobarometer 472 (2018). Sport and Physical Activity 2017. https://sport.ec.europa.eu/news/new-eurobarometer-on-sport-and-physical-activity.

[93] U.S. Department of Health and Human Services (2019). Physical Activity Guidelines for Americans.

[94] Fletcher G.F., Blair S.N., Blumenthal J., Caspersen C., Chaitman B., Epstein S., Falls H., Froelicher E.S., Froelicher V.F., Pina I.L. (1992). Statement on Exercise. Benefits and Recommendations for Physical Activity Programs for all Americans. A Statement for Health Professionals by the Committee on Exercise and Cardiac Rehabilitation of the Council on Clinical Cardiology, American Heart Association. Circulation (New York, N.Y.).

[95] Martin S.B., Morrow J.R., Jackson A.W., Dunn A.L. (2000). Variables Related to Meeting the CDC/ACSM Physical Activity Guidelines. Med. Sci. Sport. Exerc..

[96] Gregg E.W., Cauley J.A., Stone K., Thompson T.J., Bauer D.C., Cummings S.R., Ensrud K.E. (2003). Relationship of Changes in Physical Activity and Mortality among Older Women. J. Am. Med. Assoc. JAMA.

[97] Chomistek A.K., Manson J.E., Johnson K.C., Eaton C.B., Stefanick M.L., Lu B., Sands-Lincoln M., Going S.B., Garcia L., Allison M.A. (2013). Relationship of Sedentary Behavior and Physical Activity to Incident Cardiovascular Disease: Results from the Women’s Health Initiative. J. Am. Coll. Cardiol..

[98] 98. Celis-Morales C.A., Perez-Bravo F., Ibanez L., Salas C., Bailey M.E.S., Gill J.M.R. (2012). Objective Vs. Self-Reported Physical Activity and Sedentary Time: Effects of Measurement Method on Relationships with Risk Biomarkers. PloS ONE.

[99] Clemes S.A., David B.M., Zhao Y., Han X., Brown W. (2012). Validity of Two Self-Report Measures of Sitting Time. J. Phys. Act. Health.

[100] Ferrari G.L.D.M., Kovalskys I., Fisberg M., Gomez G., Rigotti A., Sanabria L.Y.C., Garcia M.C.Y., Torres R.G.P., Herrera-Cuenca M., Zimberg I.Z. (2020). Comparison of Self-Report Versus Accelerometer—Measured Physical Activity and Sedentary Behaviors and their Association with Body Composition in Latin American Countries. PLoS ONE.

[101] Sansano-Nadal O., Wilson J.J., Martín-Borràs C., Brønd J.C., Skjødt M., Caserotti P., Roqué I Figuls M., Blackburn N.E., Klenk J., Rothenbacher D. (2022). Validity of the Sedentary Behavior Questionnaire in European Older Adults using English, Spanish, German and Danish Versions. Meas. Phys. Educ. Exerc. Sci..

[102] Healy G.N., Clark B.K., Winkler E.A., Gardiner P.A., Brown W.J., Matthews C.E. (2011). Measurement of adults’ sedentary time in population-based studies. Am. J. Prev. Med..

